# Estimation of aortic pulse wave transit time in cardiovascular magnetic resonance using complex wavelet cross-spectrum analysis

**DOI:** 10.1186/s12968-015-0164-7

**Published:** 2015-07-30

**Authors:** Ioannis Bargiotas, Elie Mousseaux, Wen-Chung Yu, Bharath Ambale Venkatesh, Emilie Bollache, Alain de Cesare, Joao A.C. Lima, Alban Redheuil, Nadjia Kachenoura

**Affiliations:** Sorbonne Universités, UPMC Univ Paris 06, INSERM, CNRS, Laboratoire d’Imagerie Biomédicale, F-75013 Paris, France; INSERM, UMR 970, PARCC, F-75015 Paris, France; Department of Cardiovascular Radiology, Hôpital Européen Georges Pompidou, Paris, France; Taipei Veterans General Hospital, Taipei, Taiwan; Division of Cardiology, Johns Hopkins University, Baltimore, MD USA; Northwestern University, Feinberg School of Medicine, Department of Radiology Chicago, IL, 60611 USA; Institut de Cardiologie, Hôpital Pitié Salpêtrière, Paris, France; Imaging Core Lab, ICAN, Paris, France

## Abstract

**Background:**

Aortic pulse wave velocity (PWV), which substantially increases with arterial stiffness and aging, is a major predictor of cardiovascular mortality. It is commonly estimated using applanation tonometry at carotid and femoral arterial sites (cfPWV). More recently, several cardiovascular magnetic resonance (CMR) studies have focused on the measurement of aortic arch PWV (archPWV). Although the excellent anatomical coverage of CMR offers reliable segmental measurement of arterial length, accurate transit time (TT) determination remains a challenge. Recently, it has been demonstrated that Fourier-based methods were more robust to low temporal resolution than time-based approaches.

**Methods:**

We developed a wavelet-based method, which enables temporal localization of signal frequencies, to estimate TT from ascending and descending aortic CMR flow curves. This method (archPWV_WU_) combines the robustness of Fourier-based methods to low temporal resolution with the possibility to restrict the analysis to the reflectionless systolic upslope. We compared this method with Fourier-based (archPWV_F_) and time domain upslope (archPWV_TU_) methods in relation to linear correlations with age, cfPWV and effects of decreasing temporal resolution by factors of 2, 3 and 4. We studied 71 healthy subjects (45 ± 15 years, 29 females) who underwent CMR velocity acquisitions and cfPWV measurements.

**Results:**

Comparison with age resulted in the highest correlation for the wavelet-based method (archPWV_WU_:r = 0.84,*p* < 0.001; archPWV_TU_:r = 0.74,*p* < 0.001; archPWV_F_:r = 0.63,*p* < 0.001). Associations with cfPWV resulted in the highest correlations for upslope techniques whether based on wavelet (archPWV_WU_:r = 0.58,*p* < 0.001) or time (archPWV_TU_:r = 0.58,*p* < 0.001) approach. Furthermore, while decreasing temporal resolution by 4-fold induced only a minor decrease in correlation of both archPWV_WU_ (r decreased from 0.84 to 0.80) and archPWV_F_ (r decreased from 0.63 to 0.51) with age, it induced a major decrease for the archPWV_TU_ age relationship (r decreased from 0.74 to 0.38).

**Conclusions:**

By CMR, measurement of aortic arch flow TT using systolic upslopes resulted in a better correlation with age and cfPWV, as compared to the Fourier-based approach applied on the entire cardiac cycle. Furthermore, methods based on harmonic decomposition were less affected by low temporal resolution. Since the proposed wavelet approach combines these two advantages, it might help to overcome current technical limitations related to CMR temporal resolution and evaluation of patients with highly stiff arteries.

## Background

Aortic stiffness through interplay between causal and aggravating factors such as aging and other cardiovascular risk factors has been associated with coronary heart disease [1], as well as with cardiovascular mortality [2]. It is highly related to changes in aortic pressure waveform morphology [3] and hemodynamics. Such changes are characterized by an increase in pulse pressure and augmentation index [4], as well as by an increase in pulse wave velocity (PWV) [5]. The latter, which can be commonly derived from non-invasive carotid and femoral pressure curves, is well recognized as a strong predictor of cardiovascular events and mortality [6].

More recently, local geometric, functional and hemodynamic properties of the proximal aorta can be studied using cardiovascular magnetic resonance (CMR). Among such indices, aortic arch PWV (archPWV) is calculated from CMR as the ratio between the length of an aortic segment and the transit time required by the flow wave to travel throughout this segment. Indeed, the estimation of archPWV is feasible using CMR through plane [7–9], in-plane [10] or 4D [11] velocity data. Although aortic length estimation is highly accurate, especially when using 3D CMR measurements [11], pulse wave transit time (TT) estimation between selected aortic locations remains a challenge because of the relatively low temporal resolutions achievable by CMR, compared to tonometric techniques for example, and also due to the presence of wave reflections. Consequently, several methods have been proposed for TT measurement from CMR flow or flow velocity curves simultaneously measured from two aortic sites. Previously proposed TT estimation methods include: 1) the foot-to-foot approach inspired from conventional tonometric pressure wave analysis [5], 2) point-based methods [12] relying on a single time point such as 50 % of the systolic upslope [13, 14], systolic peak or maximal upslope acceleration [9], and 3) wave-based methods using similarity criteria such as cross correlation [15] or Fourier analysis to calculate phase differences between two given aortic locations [16].

While point-based methods have been shown to be robust on pressure waves, they suffer from the relatively low temporal resolution of CMR flow curves [7, 17]. Regarding wave-based methods, when applied to the whole cardiac cycle, they might suffer from the presence of wave reflections which develop in late systole, but even earlier in the elderly, affecting the morphology of the systolic downslope [5, 7]. To overcome these issues, recent wave-based techniques focused only on the systolic upslope of the CMR flow curve have been proposed [7, 8, 18]. These methods have been shown to be more robust to low temporal resolution, especially when performed in the frequency domain as recently reported by Meloni et al [16].

Accordingly, our aim in this study was to develop a robust TT measurement method which combines the robustness of frequency domain methods to low temporal resolution as well as the ability to restrict the analysis to the reflectionless systolic upslope. This new method is based on complex wavelet approach which enables temporal localization of signal frequencies and thus, a proper restriction of the analysis to the systolic upslope. In the latter time period, TT is calculated from the phase differences among low, medium and high harmonics. The archPWV resulting from the latter method was compared with the arch PWV obtained using time domain upslope-to-upslope [7] and Fourier-based [16] approaches, in their associations with: (i) age and (ii) tonometric carotid–femoral PWV (cfPWV). In addition, the effect of temporal resolution on the three archPWV methods was evaluated using under-sampled curves.

## Methods

We included 71 healthy volunteers (44.9 ± 14.8 years, 29 females) without overt cardiovascular disease, from two research centres using different CMR scanners (GE, 1.5 T and Siemens, 3.0 T). They underwent aortic CMR and carotid–femoral PWV as well as pressure measurements. Moreover, characteristics such as weight and height were collected. The study protocol was approved by the institutional review boards (France: Direction Recherche Clinique Assistance Publique des Hopitaux de Paris (APHP) 10.1186/s12968-015-0164-7 USA: Johns Hopkins Hospital IRB) and all subjects gave written informed consent.

### CMR acquisitions

In addition to the conventional axial and coronal SSFP data used for anatomical characterization of the aortic arch, through-plane phase contrast (PC) images of the proximal aorta were acquired for each subject in a single axial view at the level of the bifurcation of the pulmonary trunk, perpendicular to both the ascending and descending aorta to simultaneously measure flow. Acquisition planes were positioned 2 to 4 cm above the aortic junction to ensure optimal imaging quality [19] and to avoid distortion due to aortic valve motion.

The study population included two sub-groups: Group 1 included 36 subjects (43 ± 15 years, 15 females) with CMR exam on a 1.5 T scanner (Signa HDx, GEMS, Waukesha, WI, USA) at the George Pompidou European Hospital in Paris, France, and Group 2 included 35 subjects (47 ± 15 years, 14 females) with CMR exam performed on a 3.0 T scanner (Trio Tim, Siemens) at the Johns Hopkins Hospital in Baltimore, USA. CMR acquisitions were performed with cardiac phased-array coils and ECG-gated pulse sequences. For ascending and descending aortic flow measurements, a single PC slice positioned at the aforementioned axial location was acquired, using a retrospectively ECG-gated breath-hold gradient sequence with velocity encoding gradient in the through-plane direction. For the GE scanner, scan parameters were: repetition time = 7.4 ms, echo time = 3.0 ms, flip angle = 20°, views per segment = 2, rectangular field-of-view = 50 %, acquisition matrix = 256 × 128, pixel size = 1.64 mm × 1.64 mm, slice thickness = 8 mm, and encoding velocity = 200 cm/s. View sharing was used resulting in a temporal resolution of 15 ms. For the Siemens scanner, the scan parameters were: repetition time = 5.8 ms, echo time = 2.0 ms, flip angle = 30°, views per segment = 1, rectangular field-of-view = 75 %, acquisition matrix = 192 × 192, pixel size = 1.5 mm × 1.5 mm, slice thickness = 5.5 mm, and encoding velocity = 150 cm/s, number of phases per cardiac cycle was fixed to 60 phases and average temporal resolution, 20 ms.

### Applanation tonometry

Simultaneously to aortic CMR acquisitions, brachial pressure was assessed by oscillometric acquisitions (Vital Signs Monitor, Welch Allyn Inc, US) using a sensor cuff. Moreover, immediately after aortic CMR, pressure variations from right carotid and right femoral arteries were measured using applanation tonometry (for site 1 : Pulse Pen device, Diatecne, Italy. For site 2: VP-2000, Colin Corp, Japan). Carotid-femoral pulse wave velocity (cfPWV) was assessed as the ratio of the difference between the suprasternal notch-femoral and the carotid-suprasternal notch distances measured by means of a tape ruler over the body surface, to the TT measured as the foot-to-foot interval [5] between the carotid and femoral pressure waveforms.

Carotid pressure curve resulting from the average of several cardiac cycles was calibrated as previously proposed [5, 20] using the brachial mean and diastolic blood pressures measured within the magnet during CMR velocity acquisitions. Carotid systolic (SBP) and diastolic (DBP) blood pressure as well as the pulse pressure (PP) were recorded.

### CMR data analysis

**Fig. 1:**
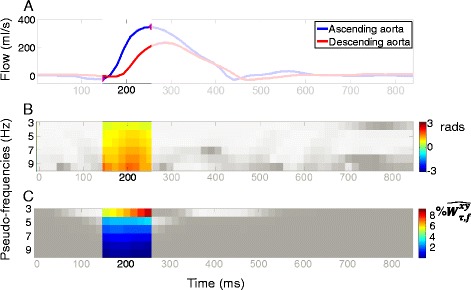
Wavelet analysis of ascending and descending aorta flow curves. Ascending and descending aortic CMR flow curves (**a**) along with the phase shift (**b**) and normalized modulus (**c**) derived by wavelet cross spectrum analysis. Systolic upslope, which is considered for the final transit time estimation, is highlighted in colour in all frames

Aortic arch length was measured three-dimensionally from sagittal oblique SSFP or dark blood T1 acquisitions as described in [7]. The 3D approach was used to take into account possible tortuosity of the aortic arch [7]. Ascending (AA) and descending (DA) aortic lumen borders were automatically detected on the modulus images for every frame of the cardiac cycle using the ARTFUN software (INSERM 1146, UPMC, Paris, France). The only manual intervention was to select the ascending and descending aortic centres and a single point on their border. The subsequent detection of the borders for the whole cardiac cycle was fully automatic and thus highly reproducible [21]. Aortic borders were then superimposed on CMR velocity images providing time varying AA and DA flow curves, which were used to automatically estimate TT by three different approaches (Fig. 1a):

### Time domain transit time (TT-TU)

Upslope method providing TT-TU as the time shift maximizing the overlap between AA and DA upslopes. This method has been previously used in several studies in the setting of aging [7] and disease [22, 23].

### Fourier-based transit time (TT-F)

Group delay method, based on Fourier analysis, and proposed recently by Meloni et al [16] was used to estimate the harmonic phase differences between AA and DA flow curves in the frequency domain. TT-F was expressed as the energy-weighted sum of harmonics phases.

1 $$ \boldsymbol{T}{\boldsymbol{T}}_{\boldsymbol{F}}=\raisebox{1ex}{${\displaystyle {\left|\boldsymbol{X}\right|}^2*\kern0.1em \boldsymbol{G}\boldsymbol{D}}$}\!\left/ \!\raisebox{-1ex}{${\displaystyle \sum {\left|\boldsymbol{X}\right|}^2}$}\right. $$

where X is the Fourier transform of ascending aortic flow x(t), |X| is its magnitude, and group delay GD, is the phase array. TT-F is expressed in seconds.

### Wavelet-based transit time (TT-WU)

A new method for TT-WU estimation was developed using Matlab (Matlab 2013, The Mathworks, Natick, MA) software. First, the wavelet cross spectrum of AA and DA aortic flow waveforms was estimated (Fig. 1), providing a time-scale distribution of cross-spectra between signals. The main advantage of this analysis is that it enables temporal localization of signal frequencies allowing us to focus only on the systolic upslope. Whereas in Fourier-analysis signals are represented as sine wave components, in wavelet analysis, signals are represented as convolutions with a set of functions derived from the scaled and shifted versions of an initial function named “mother wavelet”. Wide wavelets are shifted in time in order to detect low frequency magnitude and phase components and narrow wavelets are shifted in time in order to detect high frequency magnitude and phase components. In our case, complex Gaussian curve is used as a “mother wavelet”. AA and DA flow curves were analysed for scales which correspond to frequency components ≤ 10Hz. The frequency range (<10Hz) was chosen to minimize the effect of noise lying in high frequencies [24] and to account for the prior knowledge on systolic time period and more specifically on systolic upslope minimal duration (~100 ms). Then, complex cross spectrum (magnitude and phase difference) was calculated and the derived time-scale map was restricted to the systolic upslope $\uptau \in \left[\mathrm{Tfoot},\;\mathrm{Tpeak}\right]$ (Fig. 1). Systolic foot (Tfoot) and systolic peak (Tpeak) were automatically detected as in [7]. Then, similar to the Fourier method, group delay was estimated by the weighted sum of phase differences over harmonics ≤ 10 Hz. Further methodological details are provided in the Appendix.

For all TT estimates, local PWV (archPWV) was calculated as the ratio between aortic arch length and the estimated TT between AA and DA curves, resulting in archPWV_TU_, archPWV_F_, and archPWV_WU_. Of note, the same aortic arch length was used for the three archPWV estimates and TT estimation was fully automated and thus reproducible for the three techniques. Moreover, processing time was very low and in the same range for all techniques (<1 s for each subject). Indeed, the only source of inter-operator variation in archPWV estimation is the aortic arch length measurement which was previously [7] shown to be highly reproducible (coefficient of variation of 4 % ± 2 %).

To study effect of temporal resolution, time points of AA and DA curves were averaged by blocks of 2, 3, or 4 as previously proposed [25]. Then, for original and averaged flow curves, archPWV was calculated as the ratio between the aforementioned aortic arch length and TT values estimated fully automatically using the time domain upslope method (archPWV_TU_), the Fourier-based method (archPWV_F_) and the wavelet-based approach (archPWV_WU_).

### Statistical analysis

Continuous variables were checked for normality using the Shapiro-Wilk test and were found to be normally distributed. Associations with age and the gold standard cfPWV were studied using linear regression and correlation coefficients were provided for archPWV estimated from original and averaged AA and DA curves. To further study the effect of temporal resolution, archPWV estimated from averaged curves was compared to archPWV estimated from original AA and DA curves and linear regression slopes and correlation coefficients as well as Bland–Altman mean bias and limits of agreement, defined as mean bias ±1.96 · standard deviation, are provided. These latter analyses are performed for the overall population and then separately for both Groups 1 and 2 to assess potential effect of differences in CMR scanner and acquisition protocols. Indeed, while in Group 1 temporal resolution was fixed, in Group 2 the number of phases per cardiac cycle was fixed. For all associations, statistical significance was indicated by *p* < 0.05. Statistical analysis was performed using STATA/IC 12.0 software.

## Results

Subjects description including basic characteristics, central arterial hemodynamic and geometric properties and cfPWV are summarized in Table 1. Of note, such characteristics were provided for the whole group as well as for the two sub-groups corresponding to the two sites using different CMR scanners. Subjects of Group 2 were slightly older and had a higher BMI than those of Group 1. They also had slightly higher blood pressure, pulse pressure and cfPWV. Aortic length was similar between the two groups.

**Table 1:** Subjects characteristics

Parameters	Group 1 (*n* = 36)	Group 2 (*n* = 35)	Total (*n* = 71)
Age (years)	42.7 ± 14.9	47.2 ± 14.5	44.9 ± 14.8
BMI (kg.m^-2^)	22.8 ± 2.8	24.7 ± 4.6	23.8 ± 3.9
Carotid SBP (mmHg)	103.3 ± 16.3	114.4 ± 20.6	108.8 ± 19.3
Carotid DBP (mmHg)	67.9 ± 11.5	74.5 ± 12.2	71.2 ± 12.2
Carotid PP (mmHg)	35.4 ± 9.3	39.9 ± 12.3	37.6 ± 11.3
Aortic length (cm)	11.9 ± 2.2	11.9 ± 2.3	11.9 ± 2.2
cfPWV (m.s^−1^)	7.2 ± 2.4	8.4 ± 2.3	7.8 ± 2.4

Subjects characteristics are provided for the entire group and for both Group 1 and Group 2. SBP is systolic blood pressure, DBP is diastolic blood pressure, PP is pulse pressure and cfPWV is carotid-femoral pulse wave velocity

### Comparison of the archPWV methods

Table 2 summarizes the average values of archPWV for all methods in subjects <50 years old and subjects ≥50 years old. As expected, archPWV provided by all methods were significantly higher in subjects ≥50 years old (*p* < 0.001). Moreover, archPWV_WU_ and archPWV_TU_ were in the same range while archPWV_F_ resulted in slightly higher values for both age groups, with more pronounced difference for subjects ≥50 years old.

**Table 2:** Aortic pulse wave velocity estimated from CMR data

	archPWV_WU_ (m/s)	archPWV_TU_ (m/s)	archPWV_F_ (m/s)
<50 years old (*n* = 44)	4.1 ± 1.5	4.4 ± 1.2	5.5 ± 3.1
≥50 years old (*n* = 27)	7.8 ± 2.2	8.1 ± 2.7	11.7 ± 6.2

Averaged values of aortic pulse wave velocity estimated using wavelet-based (archPWV_WU_), time-based (archPWV_TU_ as well as Fourier-based (archPWV_F_) approach in subgroups of subjects aged <50 and ≥50 years

**Fig. 2:**
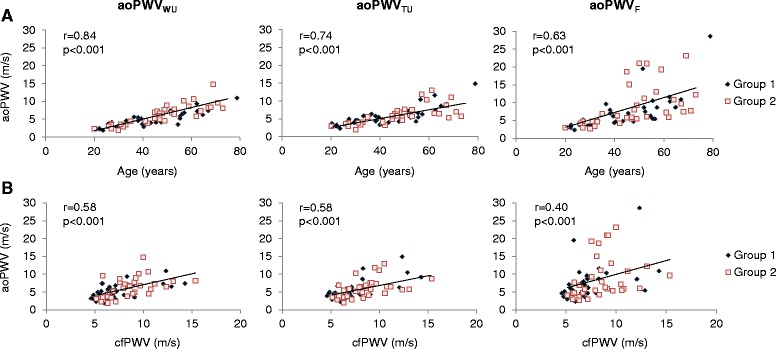
Associations with age and carotid-femoral pulse wave velocity (cfPWV). Associations between age and CMR archPWV estimated using wavelet-based (archPWV_WU_), time-based (archPWV_TU_) as well as Fourier-based (archPWV_F_) approaches (**a**). Associations between carotid-femoral pulse wave velocity (cfPWV) and CMR archPWV estimated using wavelet-based (archPWV_WU_), time-based (archPWV_TU_) as well as Fourier-based (archPWV_F_) approach (**b**). Different markers were used for subjects from Group 1 and Group 2

Figure 2a and 2b illustrate linear associations of archPWV methods with age and cfPWV, respectively. While all estimated archPWV values increased significantly with age, the wavelet-based PWV (archPWV_WU_) resulted in the highest correlation with age (r = 0.84, *p* < 0.001), compared to both the time-based method (archPWV_TU_: r = 0.74, *p* < 0.001) and the Fourier-based method (archPWV_F_: r = 0.63, *p* < 0.001). Regarding associations with cfPWV, the highest correlations were found for the upslope techniques whether based on the wavelet (archPWV_WU_: r = 0.58, *p* < 0.001) or time (archPWV_TU_: r = 0.58, *p* < 0.001) approaches. Interestingly, the Fourier-based method (archPWV_F_) which takes into account the flow curves corresponding to the whole cardiac cycle, resulted in more dispersed values among elderly individuals, even higher than cfPWV in some individuals. As illustrated in Fig. 4a, TT estimation from the whole cardiac cycle (TT-F) was subjected to the difference in time-shift between AA and DA curves during late systole as compared to early systole. Such difference resulted in shorter TT and thus in substantially high archPWV.

### Effect of temporal resolution

Table 3 shows the results of the comparisons of archPWV values estimated from the original AA and DA flow curves against those estimated from curves averaged by blocks of 2, 3 and 4, to mimic lower temporal resolution levels. While for both the wavelet and Fourier-based methods averaging had minor effect on archPWV estimation, it resulted in a pronounced alteration for the time-based method, especially at the lowest temporal resolution levels (averages by blocks of 3 or 4).

**Table 3:** Effect of temporal resolution

	Avg by 2	Avg by 3	Avg by 4
archPWV_WU_			
Linear fit slope	1.01	0.93	0.86
Bland-Altman mean bias [limits] (m/s)	0.2 [-1.45 to 1.87]	0.12 [-2.06 to 2.30]	0.10 [-2.50 to 2.71]
Correlation r(p)	0.95 (<0.001)	0.91 (<0.001)	0.87 (<0.001)
archPWV_TU_			
Linear fit slope	0.99	0.60	0.30
Bland-Altman mean bias [limits] (m/s)	0.35 [-1.72 to 2.40]	-0.07 [-4.28 to 4.22]	-0.03 [-7.81 to 7.71]
Correlation r(p)	0.88 (<0.001)	0.72 (<0.001)	0.50 (<0.001)
archPWV_F_			
Linear fit slope	1.03	1.03	0.89
Bland-Altman mean bias [limits] (m/s)	0.16 [-2.51 to 2.8]	0.42 [-3.29 to 4.12]	0.32 [-4.67 to 5.32]
Correlation r(p)	0.97 (<0.001)	0.94 (<0.001)	0.89 (<0.001)

For the three methods, aortic pulse wave velocity (archPWV) estimated from the original ascending and descending aortic flow curves, were used as reference for comparisons against archPWV estimated from curves with lower temporal resolution obtained by averaging time points by blocks of 2 (Avg by 2), 3 (Avg by 3) and 4 (Avg by 4). For these associations the linear fit slope, Bland-Altman mean bias and limits of agreement as well as linear correlation coefficients were provided. Wavelet-based approach is archPWV_WU_, time-based approach is archPWV_TU_ and Fourier-based approach is archPWV_F_

**Fig. 3:**
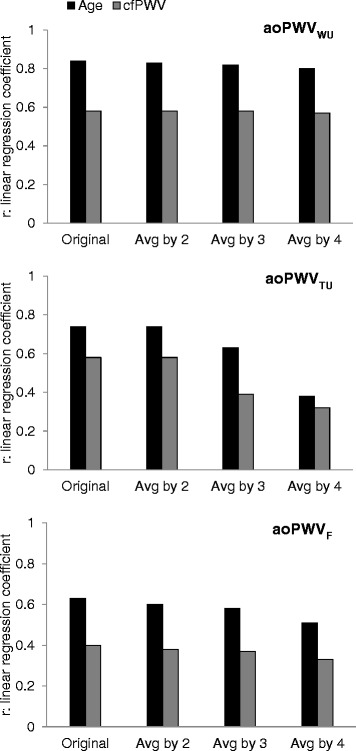
Effect of temporal resolution on correlations with age and carotid-femoral PWV (cfPWV). Correlations with age (black) and cfPWV (grey) of CMR archPWV estimated using wavelet-based (archPWV_WU_), time-based (archPWV_TU_) as well as Fourier-based (archPWV_F_) approach on the original ascending and descending aorta curves, and the subsequent averaged curves by blocks of 2 (Avg by 2), 3 (Avg by 3) and 4 (Avg by 4)

**Fig. 4:**
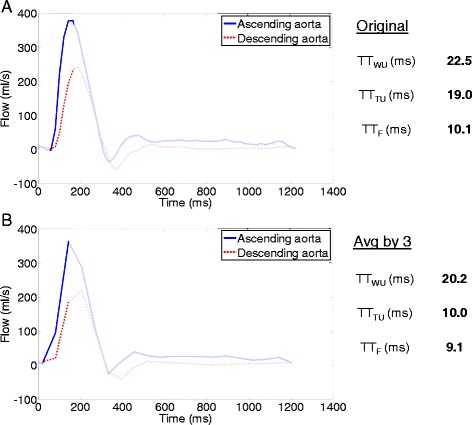
Illustration of transit time (TT) estimation and effect of lowering temporal resolution on a 59-year-old subject. **a**) Original ascending (AA) and descending (DA) aortic flow waveforms along with TT values obtained by wavelet-based (TT_WU_) and time-based (TT_TU_) approaches applied on the systolic upslope as well as by Fourier-based (TT_F_) approach applied on the entire cardiac cycle, **b**) Same waveforms averaged by blocks of 3 time points (Avg by 3) and the corresponding TT values

Similar conclusions were reached for the associations with age and cfPWV of archPWV values estimated from the original AA and DA flow curves as well as from those averaged by blocks of 2, 3 or 4 (Fig. 3). Indeed, all methods remained unchanged in terms of associations with age and cfPWV for a slight decrease in temporal resolution (averages by blocks of 2). However, after more pronounced lowering of temporal resolution (averages by blocks of 3 and 4), while the time-based method was strongly affected (Fig. 4a and 4b), archPWV based on wavelet and Fourier analysis remained fairly unchanged in terms of associations with age and cfPWV.

Finally, the analysis presented in Table 3 was performed separately for Group 1 and Group 2 and for the method based on wavelet, which was shown to be more robust to lower temporal resolution (Table 4). This analysis showed that wavelet-based archPWV behaved similarly for both CMR scanners and acquisition protocols.

**Table 4:** Effect of CMR scanner and acquisition protocol

archPWV_WU_		Avg by 2	Avg by 3	Avg by 4
Linear fit slope	Group 1	1.02	0.94	0.91
	Group 2	1.05	0.96	0.88
Bland-Altman mean bias [limits] (m/s)	Group 1	0.32 [-1.30 to 1.95]	0.30 [-1.54 to 2.16]	0.37 [-1.68 to 2.42]
	Group 2	0.07 [-1.58 to 1.73]	-0.06 [-2.51 to 2.38]	-0.16 [-3.17 to 2.84]
Correlation r(p)	Group 1	0.92 (<0.001)	0.90 (<0.001)	0.87 (<0.001)
	Group 2	0.96 (<0.001)	0.90 (<0.001)	0.85 (<0.001)

For the wavelet-based method, aortic pulse wave velocity (archPWV_WU_) estimated from the original ascending and descending aortic flow curves, was used as a reference for comparisons against archPWV_WU_ estimated from curves with lower temporal resolution obtained by averaging time points by blocks of 2 (Avg by 2), 3 (Avg by 3) and 4 (Avg by 4) in both Group 1 and Group 2

## Discussion

In this study, a new method for aortic flow TT and thus for archPWV determination from AA and DA CMR flow curves was developed and tested in 71 subjects. This multiresolution method, based on complex wavelet cross-spectrum, combines the advantages of the time domain and frequency-based methods which respectively: 1) focus the analysis on the systolic upslope to avoid wave reflections effects , and 2) take into account further harmonics to produce a more detailed evaluation of the aortic flow curves composition. The proposed method was compared against Fourier-based approach, recently proposed by Meloni et al [16], as well as the time domain upslope method, previously presented as a reliable technique for TT assessment [7], relation to age, cfPWV, and the effects of gradually lowering temporal resolution. While time (archPWV_TU_) and wavelet-based (archPWV_WU_) approaches showed equal correlation with the established cfPWV values, the wavelet-based (archPWV_WU_) method showed a higher correlation with age. Moreover, the wavelet-based and Fourier-based methods were shown to be more robust at lower temporal resolutions. Of note, the Fourier-based technique resulted in the weakest correlations with age and cfPWV.

It is well known that cfPWV is a reliable marker of arterial aging and a strong predictor of cardiac events and mortality [1, 2]. Applanation tonometry enables straightforward measurement of cfPWV which is widely used as a global and comprehensive marker of arterial stiffness and aging. More recently CMR, with its anatomic, functional and velocity-based capabilities, has been shown to provide accurate regional assessment of aortic geometry, compliance, distensibility and hemodynamics, which help disentangle physiological from pathological aspects of aortic alterations in the pathways of aging and load-related diseases. Among diverse CMR indices of aortic stiffness, PWV has been proposed in several studies as an important disease marker. A crucial advantage of CMR in PWV evaluation is its specific accuracy in the estimation of the arterial length travelled by the pulse wave [26]. However, regarding TT estimation, several techniques [5, 7–9, 12–16, 18] have been proposed but no consensual method has yet been chosen as the standard. This is mainly due to technical issues affecting such measurements as: 1) lower temporal resolution of CMR velocity data as compared to applanation tonometry pressure data, 2) time varying velocity-to-noise ratio (VNR) of CMR flow curves caused by a fixed encoding velocity throughout the cardiac cycle, 3) modification in late systolic flow waveform caused by wave reflections [5] and backward flow, which has been shown to be mainly associated with changes in aortic geometry [27].

Due to these important technical concerns, wave-based methods is preferred over point-based (peak-to-peak, foot-to-foot, maximal derivative-to-maximal derivative) methods since they are less prone to sampling error and noise [9]. However, when wave-based methods are applied to entire flow curves, they may be influenced by the effects of backward flow and wave reflections. For this reason, methods focusing on the systolic upslope [7, 8, 18, 28], which are less affected by wave reflections, provide higher correlations with age and cfPWV [7, 8]. Such strong correlations were confirmed in elderly subjects [7], in whom wave reflections and backward flow are expected to develop earlier in the cardiac cycle and be more pronounced.

Recently, TT determination using Fourier analysis has been proposed by Meloni et al [16], and has been shown to be highly robust in relation to the potential influence of low temporal resolution when compared to time domain methods.

In this study, a method combining the advantages of upslope approaches with those of frequency-based method is proposed, while using wavelet based approach which introduces both frequency and time dimensions. An alternative would be to use a variant of the fast Fourier transform, known as the short time Fourier transform (STFT). STFT performs a Fourier transform within a fixed time window that is shifted along the signal in order to provide frequency components over time. The selection of a suitable window size for effective signal decomposition depends on the frequencies of interest and also determines the lowest resolvable frequency. In addition to the general assumption of stationary signals, which is erroneous in our case, the uniformity of the time window across frequencies is a limitation of this approach. Indeed, optimal characterization of temporal changes in high-frequency signals requires shorter time windows than those needed to optimally characterize low-frequency signals. For example, if upslope duration determines the window size of STFT, the components of lower frequencies (which carry most of the signal’s energy) could not be detected and the components of higher frequencies could not be temporally located inside the window. Any attempt to non-artificially increase the frequency resolution and thus detect lower harmonics would cause an inclusion of the reflection-subjected late systolic part of the flow curve. Therefore, a multi-resolution approach like wavelet analysis is desirable since it enables: 1) window size variations across frequencies to optimize time-frequency resolution, 2) analysis of non-stationary dynamic signals.

Accordingly, we used wavelet analysis of the early systolic AA and DA upslope, to identify low, medium and high harmonic components which are present in early systole simultaneously. Complex wavelet analysis is able to locate in time the phase difference of each harmonic between the AA and DA flow curves. Complex wavelet cross spectrum analysis is a powerful technique to estimate a time-scale phase-differences and magnitude spectra [29]. Finally, TT calculation was inspired by the idea of group delay previously presented by Meloni et al [16], which relies on the magnitude weighted average of phase-shifts.

### Arch-PWV results

Pulse wave velocity measurements obtained by the wavelet-based technique (Table 2, Fig. 2) were consistent with those previously reported in the literature [7, 10, 11, 18, 30, 31] and were in the same range with values obtained using the time domain (archPWV_TU_) method (Table 2). Regarding Fourier-based method, archPWV values in some elderly subjects were higher than cfPWV, which is not consistent with physiological knowledge since PWV is expected to increase towards periphery [32]. These erroneous estimates are more pronounced in elderly subjects probably due to the presence of wave reflections and late systolic backward flow in this population. Indeed, such reflections might induce changes in late systolic flow waveforms and consequently modify the time shift between AA and DA curves, as compared to the time-shift observed in early systole (Fig. 4a).

### Effect of temporal resolution

The time domain method failed to robustly estimate archPWV after significant reductions in temporal resolution (averaging by blocks of 3 or 4), because the number of available points characterizing the curve upslope decreases drastically and the estimation of time-shift optimizing curves overlap becomes more unstable and prone to sampling error. On the other hand, both methods which operate in the Fourier and wavelet domains, were highly robust to lower temporal resolution (Table 3, Fig. 3 and Fig. 4b) corroborating the suggestions of Meloni et al [16]. Indeed, for both methods, even after averaging by blocks of 4, low frequencies which contain the majority of the flow curve energy are always included in the TT measurement. The advantage of the wavelet approach reflects also in its ability to focus the analysis on the upslope of the flow curve. The similarity of results (Table 4) obtained from Group 1 vs Group 2 indicates that the wavelet-based method remain robust despite differences in CMR magnets and acquisition protocols.

With further regards to methodological consideration, the first potential limitation of our study was the absence of comparison against invasive measurements of PWV using catheterization. However, the known physiological increase of PWV with normal aging as well as the comparison with the cfPWV gold standard were used as reliability criteria for archPWV estimates. Another potential limitation was the use of through-plane velocity data which restricted the estimation of PWV to a single aortic segment, namely the aortic arch. An alternative would have been to use in-plane or 4D CMR acquisitions which enable to capture flow velocities with large anatomical coverage (aortic arch and abdominal aorta). However, the primary purposes of our study were to develop a new method for TT calculation and to test its consistency and robustness to variation in temporal resolution. In theory, the newly proposed method for TT determination can be applied to both in-plane and 4D CMR velocity or flow curves without methodological adjustment.

## Conclusions

For CMR methods estimating TT from aortic flow curves, focusing only on systolic upslopes results in stronger correlations with age and cfPWV as compared to the Fourier-based approach which takes into account the entire cardiac cycle. In addition, while the effect of low temporal resolution was minor for methods based on the harmonic decomposition of AA and DA flow curves using either Fourier or wavelet analyses, it was more prominent for the time domain method. As a result, the application of the wavelet approach to the usplopes of AA and DA flow curves provides the strongest correlations for the associations with age and cfPWV. Thanks to its robustness to low temporal resolution, the wavelet-based approach can be of major usefulness to the analysis of CMR derived 4D flow data in the aortic arch.

## Appendix

Our work requires accurate and local harmonic decomposition of flow curves and therefore redundant time and scale analysis. Since the parsimonious coding is a secondary objective, continuous wavelet transform was preferred instead of discrete wavelet transform.

The continuous wavelet transform of a flow curve x(t) is defined as the convolution of x(t) with a set of continuous functions ψ_τ,s_(t) derived from the scaled and translated (shifted) versions of the mother wavelet ψ(t).

2 $$ {\boldsymbol{\psi}}_{\boldsymbol{\tau}, \boldsymbol{s}}\left(\boldsymbol{t}\right) = \frac{1}{\sqrt{\left|\boldsymbol{s}\right|}}\boldsymbol{\psi} \left(\frac{\boldsymbol{t}-\boldsymbol{\tau}}{\boldsymbol{s}}\right) $$

where τ corresponds to the time shift information in the wavelet transform, and s which is the scale parameter, is inversely proportional to frequency. Large scales correspond to low frequencies while low scales to high frequencies. Technically, ψ(t) must be a continuous oscillating function with a rapid decay at infinity and with zero average. The convolution of x(t) with ψ_τ,s_(t) transforms x(t) into the wavelet domain expanding x(t) into a 2-dimensional space (τ,s) with local oscillatory properties expressed relative to time:

3 $$ \begin{array}{l}{\boldsymbol{W}}^{\boldsymbol{x}}\left(\boldsymbol{\tau},\ \boldsymbol{s}\right) = {\displaystyle \underset{-\infty }{\overset{\infty }{\int }}}\boldsymbol{x}\left(\boldsymbol{t}\right){\boldsymbol{\psi}}_{\boldsymbol{\tau}, \boldsymbol{s}}^{*}\left(\boldsymbol{t}\right)\boldsymbol{d}\boldsymbol{t}\\ {} = \frac{1}{\sqrt{\left|\boldsymbol{s}\right|}}{\displaystyle \underset{-\infty }{\overset{\infty }{\int }}}\boldsymbol{x}\left(\boldsymbol{t}\right){\boldsymbol{\psi}}^{*}\left(\frac{\boldsymbol{t}-\boldsymbol{\tau}}{\boldsymbol{s}}\right)\boldsymbol{d}\boldsymbol{t}\end{array} $$

where * denotes a complex conjugate. In continuous wavelet analysis, wavelet families with an explicit definition formula, such as Gaussian, are more suitable instead of wavelet with scaling functions, such as Daubechies, Biorthogonal, Symlets and Coiflet, which are designed for discrete wavelet analysis and faster decompositions [33]. For phase extraction of real signals, the complex continuous wavelet decomposition is more suitable compared to the real continuous or discrete wavelet analysis. Therefore, the complex Gaussian wavelet shown below has been selected:

4 $$ \boldsymbol{\psi} \left(\boldsymbol{t},\boldsymbol{n}\right)={\boldsymbol{C}}_{\boldsymbol{n}}{\boldsymbol{e}}^{\boldsymbol{it}}{\boldsymbol{e}}^{-{\boldsymbol{t}}^2} $$

where in this work n was set equal to 4 and corresponds to the 4^th^ order derivative of the function above. C_n_ is an appropriate constant for which the 2^nd^ norm of the 4^th^ derivative of ψ(t,n) is equal to 1. The choice of the 4^th^ derivative was based on the time and frequency resolution trade-off since higher order would be poor in temporal resolution while lower order would be poor in frequency resolution.

Scales (s) are automatically calculated for every subject, depending on f_c_ and time sampling period (ΔΤ) of the flow signals. Chosen scales (s_1_ ≤ s < s_10Hz_) correspond to the ceiling of the combination between central frequency, flow curves sampling period and the desired pseudo-harmonics (f_1_ ≤ f_s_ < 10Hz), where f_s_ was calculated using the formula below and f_1_ corresponds to the fundamental frequency of systolic duration. The frequency range (<10Hz) was chosen to minimize the effect of noise lying in high frequencies [24] and to account for the prior knowledge on systolic time period and more specifically on systolic upslope minimal duration (~100 ms).

5 $$ \boldsymbol{s}=\frac{{\boldsymbol{f}}_{\boldsymbol{c}}}{{\boldsymbol{f}}_{\boldsymbol{s}}\times \boldsymbol{\varDelta} \boldsymbol{T}} $$

Given the wavelet transforms (W^x^ and W^y^) of two signals x(t) and y(t) which in this study correspond to the AA and DA flow curves, complex cross spectrum was estimated as:

6 $$ {\boldsymbol{W}}^{\boldsymbol{x}\boldsymbol{y}}\left(\boldsymbol{\tau},\ \boldsymbol{s}\right)={\boldsymbol{W}}^{\boldsymbol{x}}\left(\boldsymbol{\tau}, \boldsymbol{s}\right){\boldsymbol{W}}^{\boldsymbol{y}}{\left(\boldsymbol{\tau}, \boldsymbol{s}\right)}^{*} $$

Such estimation resulted in a complex time-scale map which is afterwards converted to a time-pseudo-frequency map. Then, similar to the Fourier method, group delay was estimated by the weighted sum of phase differences over the first harmonics, up to 10 Hz, and within the time interval corresponding to the systolic upslope $\uptau \in \left[\mathrm{Tfoot},\;\mathrm{Tpeak}\right]$. Systolic foot and systolic peak were automatically detected as in [7].

7 $$ \boldsymbol{T}{\boldsymbol{T}}_{\boldsymbol{W}\boldsymbol{U}}={\displaystyle \sum_{\boldsymbol{\tau} =\boldsymbol{Tfoot}}^{\boldsymbol{T}\boldsymbol{peak}}}{\displaystyle \sum_{\boldsymbol{f}={\boldsymbol{f}}_1}^{10}}\widehat{{\boldsymbol{W}}_{\boldsymbol{\tau}, \boldsymbol{f}}^{\boldsymbol{xy}}}\bullet \frac{\boldsymbol{\varphi} \left({\boldsymbol{W}}_{\boldsymbol{\tau}, \boldsymbol{f}}^{\boldsymbol{xy}}\right)}{\boldsymbol{f}\bullet 2\boldsymbol{\pi}}\kern1em \mathrm{with}\kern1.25em \widehat{{\boldsymbol{W}}_{\boldsymbol{\tau}, \boldsymbol{f}}^{\boldsymbol{xy}}}=\frac{{\boldsymbol{W}}_{\boldsymbol{\tau}, \boldsymbol{f}}^{\boldsymbol{xy}}}{{\displaystyle \sum}\left|{\boldsymbol{W}}_{\boldsymbol{\tau}, \boldsymbol{f}}^{\boldsymbol{xy}}\right|} $$

where the factor $\frac{\boldsymbol{\varphi} \left({\boldsymbol{W}}_{\boldsymbol{\tau}, \boldsymbol{f}}^{\boldsymbol{xy}}\right)}{\boldsymbol{f}\bullet 2\boldsymbol{\pi}}$ is the phase difference map converted to TT map.
